# Chronic kidney disease of uncertain aetiology: prevalence and causative factors in a developing country

**DOI:** 10.1186/1471-2369-14-180

**Published:** 2013-08-27

**Authors:** Nihal Jayatilake, Shanthi Mendis, Palitha Maheepala, Firdosi R Mehta

**Affiliations:** 1 Ministry of Health, Colombo, Sri Lanka; 2 Management of Noncommunicable Diseases, World Health Organization, Geneva, Switzerland; 3 World Health Organization, Colombo, Sri Lanka

**Keywords:** Arsenic, Cadmium, Chronic kidney disease, Kidney disease of uncertain aetiology, Heavy metals, Lead, Pesticides

## Abstract

**Background:**

This study describes chronic kidney disease of uncertain aetiology (CKDu), which cannot be attributed to diabetes, hypertension or other known aetiologies, that has emerged in the North Central region of Sri Lanka.

**Methods:**

A cross-sectional study was conducted, to determine the prevalence of and risk factors for CKDu. Arsenic, cadmium, lead, selenium, pesticides and other elements were analysed in biological samples from individuals with CKDu and compared with age- and sex-matched controls in the endemic and non-endemic areas. Food, water, soil and agrochemicals from both areas were analysed for heavy metals.

**Results:**

The age-standardised prevalence of CKDu was 12.9% (95% confidence interval [CI] = 11.5% to 14.4%) in males and 16.9% (95% CI = 15.5% to 18.3%) in females. Severe stages of CKDu were more frequent in males (stage 3: males versus females = 23.2% versus 7.4%; stage 4: males versus females = 22.0% versus 7.3%; *P* < 0.001). The risk was increased in individuals aged >39 years and those who farmed (chena cultivation) (OR [odds ratio] = 1.926, 95% CI = 1.561 to 2.376 and OR = 1.195, 95% CI = 1.007 to 1.418 respectively, *P* < 0.05). The risk was reduced in individuals who were male or who engaged in paddy cultivation (OR = 0.745, 95% CI = 0.562 to 0.988 and OR = 0.732, 95% CI = 0.542 to 0.988 respectively, *P* < 0.05). The mean concentration of cadmium in urine was significantly higher in those with CKDu (1.039 μg/g) compared with controls in the endemic and non-endemic areas (0.646 μg/g, *P* < 0.001 and 0.345 μg/g, *P* < 0.05) respectively. Urine cadmium sensitivity and specificity were 70% and 68.3% respectively (area under the receiver operating characteristic curve = 0.682, 95% CI = 0.61 to 0.75, cut-off value ≥0.397 μg/g). A significant dose–effect relationship was seen between urine cadmium concentration and CKDu stage (*P <* 0.05). Urine cadmium and arsenic concentrations in individuals with CKDu were at levels known to cause kidney damage. Food items from the endemic area contained cadmium and lead above reference levels. Serum selenium was <90 μg/l in 63% of those with CKDu and pesticides residues were above reference levels in 31.6% of those with CKDu.

**Conclusions:**

These results indicate chronic exposure of people in the endemic area to low levels of cadmium through the food chain and also to pesticides. Significantly higher urinary excretion of cadmium in individuals with CKDu, and the dose–effect relationship between urine cadmium concentration and CKDu stages suggest that cadmium exposure is a risk factor for the pathogensis of CKDu. Deficiency of selenium and genetic susceptibility seen in individuals with CKDu suggest that they may be predisposing factors for the development of CKDu.

## Background

The study reported here describes an apparently new form of chronic kidney disease, which cannot be attributed to diabetes, hypertension or other known aetiologies, that has emerged in the North Central region of Sri Lanka [1]. Chronic kidney disease of uncertain aetiology (CKDu) is slowly progressive, probably starting in the second decade of life, and asymptomatic until advanced. Peripheral oedema and hypertension are late features. The main histopathological features include tubular atrophy, interstitial mononuclear cell infiltration and interstitial fibrosis [2]. These histological features suggest that nephrotoxins play a key role in the aetiology of CKDu.

Potential nephrotoxins are widely distributed in the environment. Exposure to environmental and other nephrotoxins, such as herbal medicines and analgesics, are known to play a role in the aetiology of chronic kidney disease. Defining their exact role in the aetiology of kidney disease is a challenge. Environmental toxins implicated in kidney damage include heavy metals, such as arsenic, cadmium, lead and uranium; mycotoxins produced by fungi in improperly stored foods; air pollutants, such as tobacco smoke; and pesticides, such as chlorpyriphos, diazinon and propanil [3-9].

Over the last 8 years, several studies have been carried out to determine the prevalence, nature and causes of CKDu in Sri Lanka [10-20]. These studies include hospital-based reviews of case series, in which high-risk areas in North Central Province were identified. The hospital studies gave rise to population-based surveys, which showed that the populations affected by CKDu are scattered in the North Central region of the country [14]. Some studies have reported the point prevalence of CKDu to be about 2–3% among those above 18 years of age [20]. Case–control and cross-sectional studies have provided some insight into associations with the condition. Specific evaluations of exposure to organophosphate and mycotoxins have been conducted. In a cross-sectional study, there was evidence of greater inhibition of acetyl cholinesterase among patients with chronic renal dysfunction in areas of high prevalence of CKDu [17]. Ochratoxin, a naturally occurring mycotoxin with nephrotoxic properties, was not found to be a contaminant of food in the region [13,16]. Some studies have shown high levels of environmental cadmium, lead, aluminium and fluoride in regions with high rates of the condition [14,15,17,19].

Recognising the gravity of the public health threat caused by CKDu, in 2010, the Ministry of Health Sri Lanka, in collaboration with the World Health Organization (WHO), launched a national research project with the aim of investigating the prevalence and aetiology of CKDu in Sri Lanka, with a view to developing appropriate preventive strategies.

The objectives of this study were to: determine the prevalence of and identify the risk factors for CKDu; compare CKDu cases and controls with regard to exposure to heavy metals/metalloids and pesticides; and analyse food, water, soil, fertilizers and weedicides for heavy metals, in order to determine whether the levels are above stipulated reference values.

**Figure 1:**
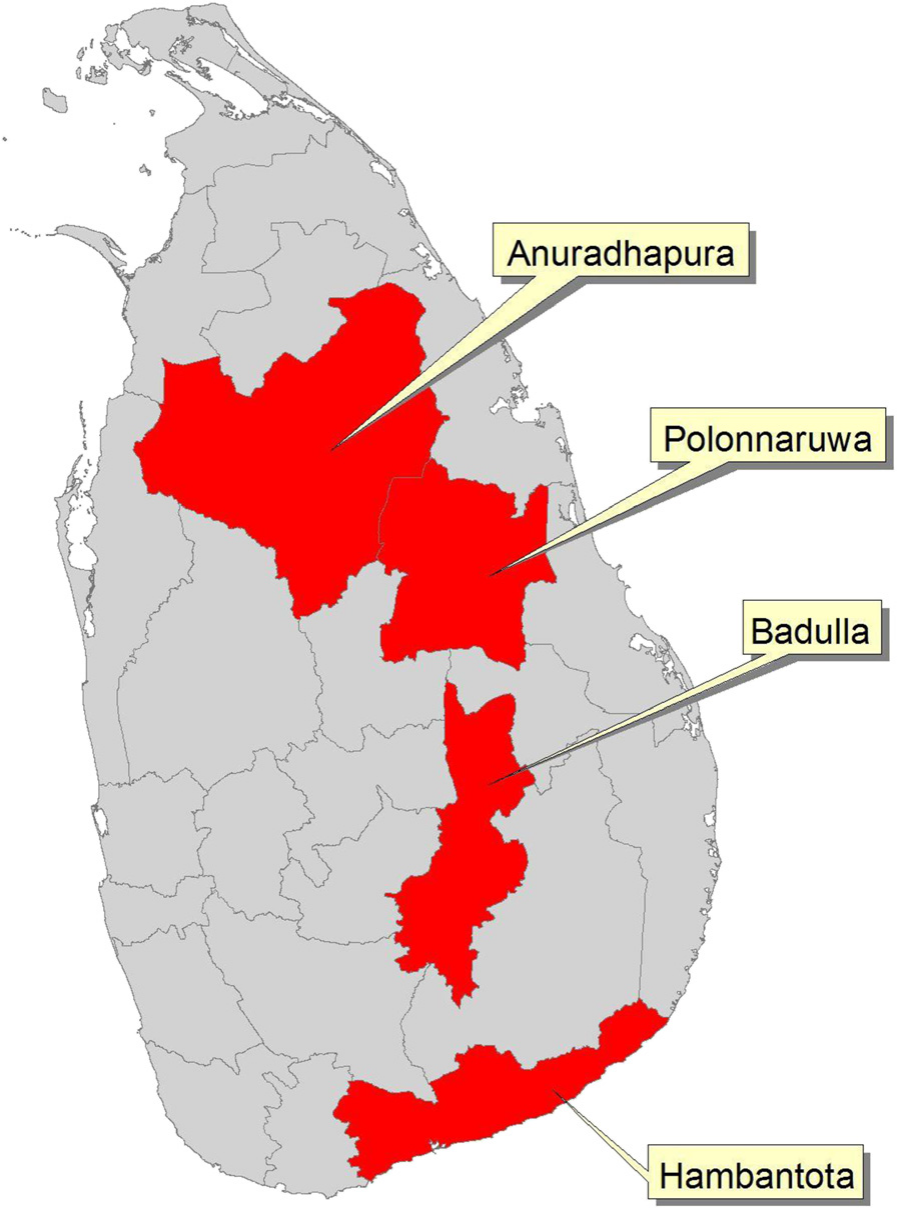
Map of Sri Lanka, showing the location of Anuradhapura, Polonnaruwa and Badulla districts, in the endemic area, and Hambantota district, in the non-endemic area.

**Figure 2:**
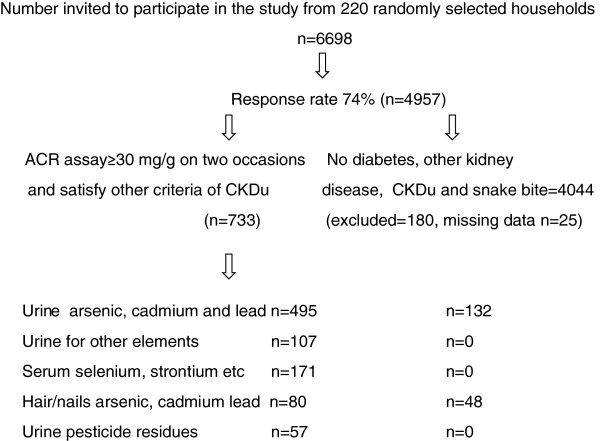
**Flow chart of the study conducted in three districts (Anuradhapura, Polonnaruwa and Badulla) in the endemic area.** ACR = albumin–creatinine ratio.

A population prevalence study was conducted in three districts in the endemic area over the period 2010–2012 (Figures 1 and 2). Arsenic, cadmium, lead and other metals, elements and pesticide residues that are potential nephrotoxins [6-9,21-24], were analysed in biological samples. The results from individuals with CKDu were compared with those from controls in the endemic area and a non-endemic area (Hambantota where CKDu has not been reported). The demographic characteristics of the three groups are shown in Table 1. All individuals with CKDu (*n* = 733) had a blood pressure below 160/100 mmHg; 6.8% of these individuals were on treatment for high blood pressure and had a blood pressure below <140/90 mmHg. Three-quarters of those who were on treatment were on angiotensin-converting enzyme inhibitors. Others were on calcium channel blockers, beta-blockers or diuretics, either alone or in combination with angiotensin-converting enzyme inhibitors.

**Table 1:** Demographic characteristics of CKDu cases in the endemic area, controls from the endemic area and controls from the non-endemic area

**Characteristic**	**Endemic area**		**Non-endemic area**
	**CKDu cases**	**Controls**	**Controls**
Total number	733	4044	250
Males, %	37.1	42.5	56
Age (years), mean (SD)	39.1 (14.2)	43.7 (13.9)	35.5 (14.0)
Farmer, %	38	43.9	18.3
BMI, kg/m^2^, mean (SD)	22.3 (4.6)	21.1 (4.1)	21.7 (4.4)
ACR ≥30 mg/g	733	0	0

*ACR* albumin–creatinine ratio, *BMI* body mass index, *SD* standard deviation.

In both endemic and non-endemic areas, water, food, tobacco, soil and agrochemicals were also analysed for heavy metals and metalloids, to determine whether they were within stipulated reference levels.

## Methods

### Case definition

The following case definition of CKDu was used. Participants who had persistent albuminuria, i.e. albumin–creatinine ratio (ACR) ≥30 mg/g in an initial urine sample and at a repeat visit, were considered to have CKDu if they satisfied the following criteria:

•no past history of glomerulonephritis, pyelonephritis, renal calculi or snake bite

•not on treatment for diabetes

•normal glycosylated haemoglobin (HbA_1c_; <6.5%)

•if on treatment for hypertension, blood pressure below <140/90 mmHg; if not on treatment for hypertension, blood pressure below <160/100 mmHg.

CKDu was graded as follows:

•*Stage 1*: persistent albuminuria (i.e. ACR ≥30 mg/g in initial and repeat urine sample) and estimated glomerular filtration rate (eGFR), using the Chronic Kidney Disease Epidemiology collaboration (CKD-EPI) equation [25] >90 ml/min/1.73 m^2^

•*Stage 2*: persistent albuminuria and eGFR 60–89 ml/min/1.73 m^2^

•*Stage 3*: persistent albuminuria and eGFR 30–59 ml/min/1.73 m^2^

•*Stage 4*: persistent albuminuria and eGFR <30 ml/min/1.73 m^2^.

### Population prevalence study

Ethical approval for the study was obtained from the Ethical Review Committee of the Sri Lanka Medical Association. All participants gave written consent for the study.

Six divisional secretariat areas (administrative divisions) were selected randomly from three districts in the endemic area. Twenty-two villages (Grama Niladari areas) were selected randomly from the six divisions. Using the electoral lists, 100 households from each village were randomly selected for the study. Males and females aged between 15 and 70 years (*n* = 6698), with no diagnosed diabetes, were invited to participate and 74% responded (Figure 2, flow diagram). Trained interviewers used a survey questionnaire to gather information on age, sex, marital status, education, occupation, smoking, alcohol consumption, current residence, duration of residence in the study area, source of drinking water, storage containers for drinking water, exposure to agrochemicals, history of snake bite, glomerulonephritis, pyelonephritis, renal calculi, use of medications including herbal medicines, and past medical history. Height was measured to the nearest 0.1 cm. Weight was measured to the nearest 0.1 kg, using a calibrated weighing scale. Participants wore light clothes and no shoes. A medical officer verified the medical information gathered and measured the blood pressure after 15 minutes’ rest, using a mercury sphygmomanometer. The average of two readings taken 5 minutes apart was used. Urine ACR, HbA_1c_ and serum and urine creatinine concentrations were also measured.

### Analytical studies

#### Arsenic, cadmium and lead in urine, blood, hair and nails

Arsenic, cadmium and lead concentrations were analysed in urine in a randomly selected subset of CKDu cases (*n* = 495) and randomly selected matched controls from the endemic area, as well as from the non-endemic area (*n* = 250).

Urine sodium, potassium, calcium, magnesium, copper, zinc and titanium concentrations were analysed in a randomly selected subset of CKDu cases (*n* = 148). Their serum was also analysed for selenium, aluminium, strontium and chromium. Hair and nail samples were analysed for cadmium, arsenic and lead in a subset of CKDu cases (*n* = 80) and controls from the endemic area (*n* = 48).

Urine samples from CKDu cases (*n* = 57) and controls from the non-endemic area (*n* = 39) were analysed for pesticide residues (2,4-D, 2,4,5-T, 2,4,5-trichlorophenol, isopropoxyphenol, pentachlorophenol, 3,5,6-trichloropyridinol, *p*-nitrophenol, 1-naphthol, 2-naphthol, glyphosate, aminomethylphosphonic acid (AMPA)).

### Arsenic, cadmium and lead in water, food, tobacco, pasture, weeds, soil, fertilizer, weedicides and pesticides

Arsenic, cadmium and lead were analysed in samples (*n* = 234) obtained from endemic and non-endemic areas. They comprised 99 sources of drinking water for individuals with CKDu (from ground wells, tube wells and natural springs), 123 other sources of water (from ground wells, tube wells, irrigation canals, reservoirs, natural springs) from the endemic area, and 12 from the non-endemic area.

Rice; pulses; vegetables, including leafy vegetables, coconut, yams and roots (e.g. kohila, lotus); freshwater fish; tobacco; pasture; and weeds obtained from endemic (*n* = 119) and non-endemic (*n* = 32) areas were analysed for arsenic, cadmium and lead.

Soil, phosphate fertilizer, pesticides and weedicides were analysed for arsenic, cadmium and lead. Soil samples were obtained from paddy fields, other types of cultivations, and reservoirs in the endemic (*n* = 88) and non-endemic (*n* = 41) areas.

### Specimen handling and analysis

Samples were collected in uncontaminated collection vials and stored frozen (−20°C) until transfer to the laboratory. All analyses were performed in a contract laboratory (Laboratory of Pathophysiology of the University of Antwerp, Belgium), which has an external quality control scheme for analysis of trace elements.

Measurements of arsenic, cadmium, lead and other elements in urine, water, vegetables, agrochemicals and soil, was performed by inductively coupled plasma mass spectrometry (MS).

Serum analyses were performed by electrothermal atomic absorption spectrometery. Limits of detection for aluminium, strontium, chromium and selenium were 0.1 μg/l, 0.5 μg/l, 0.01 μg/l and 1 μg/l respectively.

### Determination of pesticide residues in urine

Samples were shipped in dry ice and stored at −18°C until analysis. Analysis used validated liquid chromatography with tandem MS (LC-MS/MS), gas chromatography-mass spectroscopy (GC-MS) and gas chromatography with tandem mass spectrometry (GC-MS/MS) methods.

Further details of sample preparation and analytical techniques are provided in the Additional file 1.

### Statistics

The normality of data distribution was assessed with histograms. All data on metals had skewed distributions. After removal of a small number of outliers, log-transformations were used to normalise the data, in order to conduct statistical analyses. The mean, median, minimum and maximum values are reported on original data; *t*-tests of log-transformed values were used to test differences in quantitative variables. The results were also confirmed by non-parametric Wilcoxon rank-sum test.

A multiple logistic regression model was fitted for the CKDu definition. The model incorporated characteristics of interest, including age, sex, education, smoking, illicit alcohol consumption, occupation, type of agriculture, years of agriculture, source of drinking water, drinking water from paddy fields, exposure to fertilizer, exposure to weedicides and pesticides, type of water container, whether using protection against agrochemicals, and months living in the district. All were entered as categorical variables, except months living in the district. These data analyses were performed using Stata 11 and *P* values of less than 0.05 were considered statistically significant. A receiver-operating characteristic (ROC) curve was used to calculate the area under the ROC curve (AUC), to determine the cut-off values for cadmium and selenium with the best sensitivity and specificity. A multinomial logistic regression was used to assess the dose–effect relationship between metal exposure and the outcome CKDu grade. The analyses were adjusted for age and sex.

## Results

### Population prevalence study

The age-standardised prevalence of CKDu was higher in females 16.9% (95% confidence interval [CI] = 15.5% to 18.3%) than in males 12.9% (95% CI = 11.5% to 14.4%; *P* = 0.001). About 37% of those with CKDu were male. The distribution of CKDu stages 1 to 4 in males was 27.0%, 27.9%, 23.2% and 22.0% and in females 53.3%, 32.0%, 7.4% and 7.3%, respectively. More severe stages of CKDu were seen more frequently in males (stage 3: males versus females = 23.2% versus 7.4%; stage 4: males versus females = 22.0% versus 7.3%; *P* < 0.001). In both sexes, the prevalence increased with increasing age (*P* < 0.001). The prevalence in the three districts was 15.1% in Anuradhapura, 20.6% in Polonnaruwa and 22.9% in Badulla.

There was a family history of kidney disease in parents or siblings in 20% of individuals with CKDu; 2.1% of individuals with CKDu had a history of ischaemic heart disease and/or cerebrovascular disease; 0.4% had a history of long-term use of herbal medicines for hypertension; 1.8% had a history of long-term use of aspirin; and 0.6% had a history of long-term use of analgesics. Being male reduced the risk of CKDu (odds ratio [OR] = 0.745, 95% CI = 0.562 to 0.988, *P* < 0.05), and being older than 39 years increased the risk of CKDu (OR = 1.926, 95% CI = 1.561 to 2.376, *P* < 0.001). When separate logistic regressions were run for each potential exposure, only occupation type (being a chena cultivation farmer increased the OR by 19.5%) and type of agriculture (engaging in paddy cultivation compared to cultivation of vegetables and other crops [chena cultivation] decreased the OR by 26.8%) were significant (Table 2).

**Table 2:** Summary results of logistic regression analysis for exposures

**Exposure**		**95% CI**			
	**OR**	**Lower**	**Upper**	***P *value**	***n***
Education					
No education	Reference				174
School grades 1–9	0.900	0.612	1.323	0.594	4374
Higher	1.201	0.588	2.452	0.614	74
Smoking					
Never	Reference				3480
Current/former	1.072	0.813	1.415	0.619	1126
Illicit alcohol consumption					
Never	Reference				3701
Occasional/frequent/past	1.184	0.905	1.548	0.216	874
Occupation					
Other	Reference				2816
Farmer	1.195	1.007	1.418	0.041	1780
Agriculture type					
Non-paddy	Reference				315
Paddy	0.732	0.542	0.988	0.042	2620
Years working in agriculture					
<10	Reference				660
10–19	0.834	0.603	1.152	0.271	777
20–49	1.092	0.777	1.535	0.611	1182
≥50	1.322	0.462	3.785	0.602	22
Source of drinking water					
Not well	Reference				798
Well	0.971	0.785	1.202	0.793	3819
Water storage container					
Others	Reference				1741
Aluminium	1.03	0.87	1.22	0.715	2879
Protection from agrochemicals					
Yes	Reference				191
No	1.011	0.661	1.546	0.959	4271

Separate logistic regressions have been run per exposure variable; OR < 1 means protective, and OR > 1 means that the exposure increases the odds of CKDu. The total number (*n*) of observations varies per exposure, owing to missing data. All results are adjusted for sex and age. For all analyses, male sex was found to be protective and the risk increased with age.

*CI* confidence interval, *OR* odds ratio.

Being male reduced the risk of CKDu (OR = 0.745, 95% CI = 0.562 to 0.988; *P* < 0.05), and being >39 years increased the risk of CKDu (OR = 1.926, 95% CI = 1.561 to 2.376, *P* < 0.001).

### Arsenic, cadmium, lead and other elements in urine

**Figure 3:**
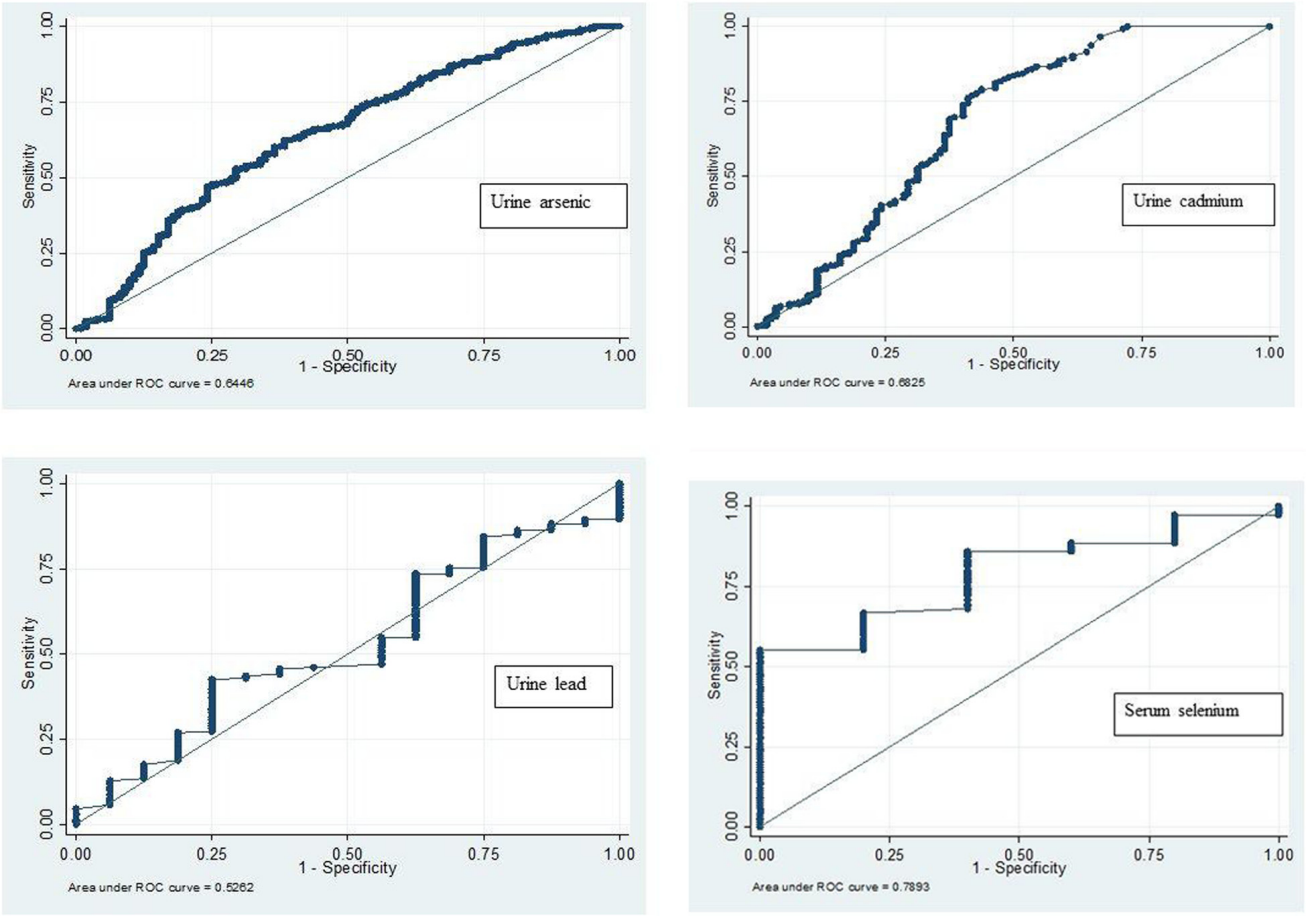
ROC curves generated with urine arsenic, cadmium and lead and serum selenium concentrations.

In CKDu cases, the concentration of cadmium in urine was significantly higher compared to controls, in both the endemic and the non-endemic areas (Table 3). Among CKDu cases, the concentration of cadmium in urine was positively correlated with lead (*r* = 0.62, *P* < 0.001) and arsenic concentrations in urine (*r* = 0.28, *P* < 0.001). There was no significant difference in urine arsenic and lead concentrations in CKDu cases compared to controls. The sensitivity and specificity for concentrations of cadmium in urine were 80% and 53.6% respectively (AUC = 0.682, 95% CI = 0.61 to 0.75, cut-off value ≥0.23 μg/g; Figure 3). At a cut-off value of ≥0.397 μg/g, sensitivity was 70% and specificity 68.3%. The sensitivity and specificity for the concentration of arsenic in urine were 90% and 23.2% respectively (AUC = 0.64, 95% CI = 0.58 to 0.71, cut-off value ≥88.57 μg/g). The concentration of lead in urine was a poor predictor of CKDu (AUC = 0.53, 95% CI 0.38 to 0.67). Dose–response analysis showed that cadmium exposure is a risk factor for the development of CKDu: *P* = 0.019 for stage 3 and *P* = 0.024 for stage 4. There was no significant dose–effect relationship between the concentration of arsenic, lead or selenium in urine and the stage of CKDu.

**Table 3:** Urine concentration of arsenic, cadmium and lead for CKDu cases compared with controls from the endemic and non-endemic areas

	**Mean, median (range) of concentration in urine (μg/g creatinine)**		
	**Arsenic**	**Cadmium**	**Lead**
CKDu cases (*n* = 495)	45.447, 26.3 (0.4 to 616.6)	1.039, 0.695 (0.005 to 8.93)	1.153, 0.95 (0.04 to 8.53)
Controls from endemic area (*n* = 132)	92.443, 6.99 (0.2 to 966.29)	0.646, 0.18, (0.005 to 5.13)^a^	1.254, 0.793 (1.21 to 6.64)
Controls from non-endemic area (*n* = 250)	56.572, 42.025 (5.38 to 350.28)	0.345, 0.265 (0.005 to 2.079)^b^	2.099, 1.434 (0.277 to 20.9)

^a^ Urine cadmium concentration of cases compared to controls from endemic area *P* < 0.001.

^b^ Urine cadmium concentration of cases compared to controls from non-endemic area *P* < 0.05.

Urine concentrations of sodium, potassium, calcium, magnesium, copper, zinc, and titanium in CKDu cases were within normal limits (Additional file 2).

### Serum aluminium, chromium, selenium and strontium in CKDu cases

Serum aluminium and chromium levels were within normal limits (Additional file 2). Serum selenium levels in subjects with CKDu ranged from 50.0 μg/l to 121.8 μg/l (reference range = 54 μg/l to 163 μg/l). A serum selenium concentration of 90 μg/l is required to reach the maximum level of glutathione peroxidise [26]. About two-thirds (63%) of subjects had selenium levels below this cut-off value. Serum strontium levels were above normal limits (mean = 83.17 μg/l, standard deviation [SD] = 32.15 μg/l; reference range = 14 μg/l to 84 μg/l). The sensitivity and specificity for serum selenium were 80% and 60% respectively (AUC = 0.789, cut = off value ≥94.3 μg/l; Figure 3).

### Cadmium and arsenic in hair and nails

A significantly higher cadmium concentration was also seen in the nails of CKDu cases (*n* = 80, mean = 0.017 μg/g, median = 0.007 μg/g, minimum = 0.001 μg/g, maximum = 0.347 μg/g) compared to controls (*n* = 48) from the endemic area (mean = 0.009 μg/g, median = 0.001 μg/g, minimum = 0.001 μg/g, maximum = 0.091 μg/g; *P* < 0.05).

Arsenic levels in hair were significantly higher in CKDu cases (*n* = 80; mean = 0.144 μg/g, median = 0.139 μg/g, minimum = 0.00 μg/g, maximum = 0.452 μg/g), compared to controls (*n* = 48) from the endemic area (mean = 0.125 μg/g, median = 0.103 μg/g, minimum = 0.006 μg/g, maximum = 1.214 μg/g; *P* < 0.05).

### Arsenic, cadmium, lead and uranium in water (endemic area *n* = 222, non = endemic area, *n* = 12)

**Figure 4:**
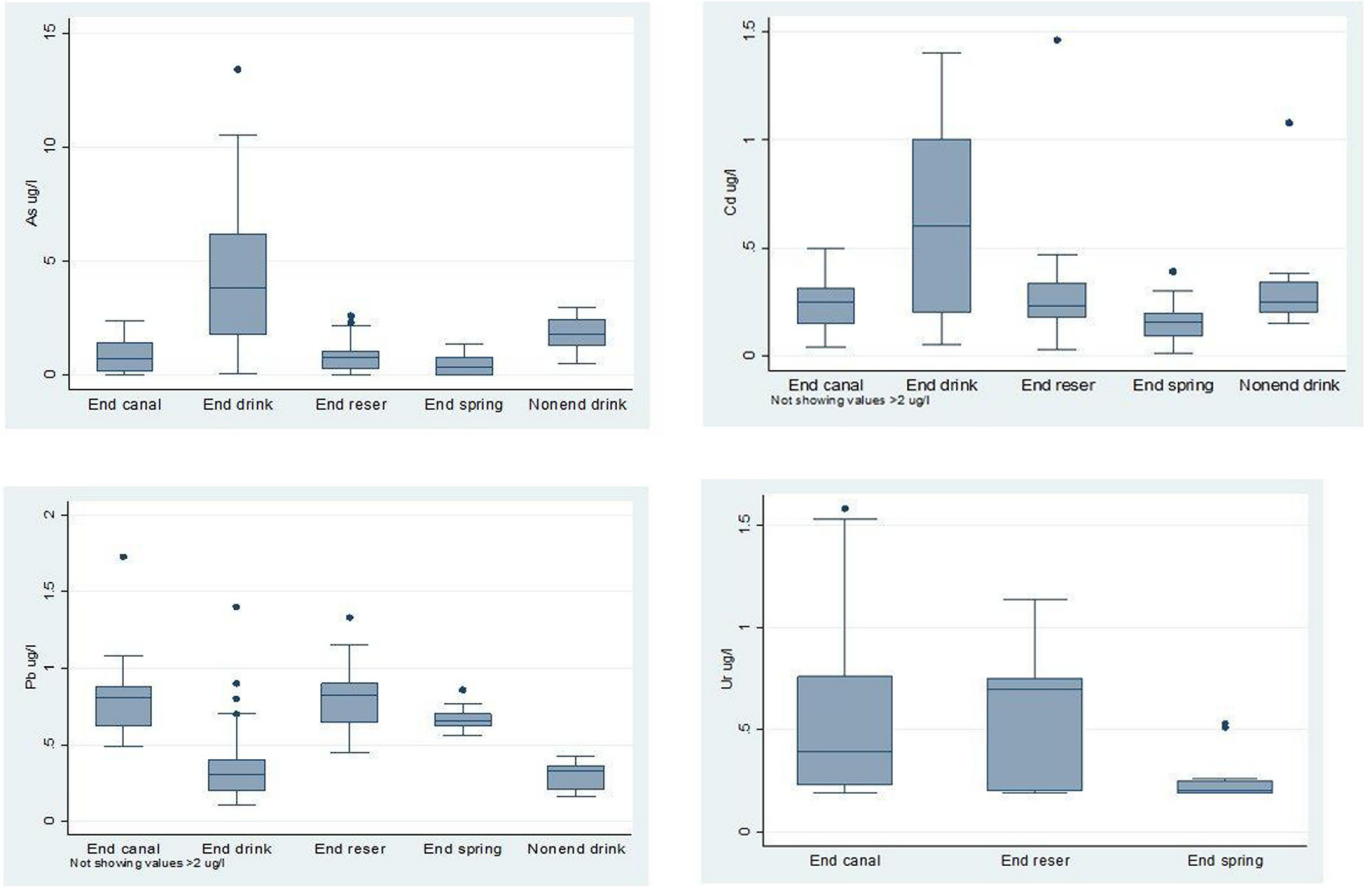
**Concentration of arsenic, cadmium, lead and uranium in water**^**a **^**in the endemic area (*n*** **= 222) and non-endemic area (*n*** **= 12).** End canal = endemic area canal; End drink = endemic area drinking water; End reser = endemic area reservoir; End spring = endemic area spring; nonend drink = non-endemic area drinking water. Horizontal lines within the boxes represent the median values. The ends of the solid lines extending on either side of the boxes represent the minimum and the maximum. The dark dots are outliers; defined as being more than 1.5 interquartile ranges away from the box. The interquartile range is the distance between the upper part of the box and the lower part of the box. ^a^ Reference limits: arsenic <10 μg /l, cadmium <3 μg/l, lead <10 μg/l, uranium <2 μg/l [21].

Levels of cadmium, lead and uranium in sources of drinking water (Figure 4) used by individuals with CKDu (*n* = 99) were within normal limits. Arsenic was borderline or raised in four samples (9.9 μg/l, 10.2 μg/l, 10.5 μg/l, 13.4 μg/l). Repeat analysis (*n* = 32) from the four sources showed normal arsenic levels.

In water samples from other sources, the arsenic concentration was 22.2 μg/l and 9.8 μg/l in two samples taken from a canal and a reservoir, the cadmium concentration was 3.46 μg/l in one sample from a reservoir and the lead concentration was 12.3 μg/l in one sample from a reservoir in the endemic area. All other samples from wells, tube wells, irrigation canals, pipe-borne water, reservoirs and natural springs, including those taken from the non-endemic area, had normal arsenic, cadmium and lead levels.

### Arsenic, cadmium and lead in food, tobacco leaves, pasture and weeds

**Figure 5:**
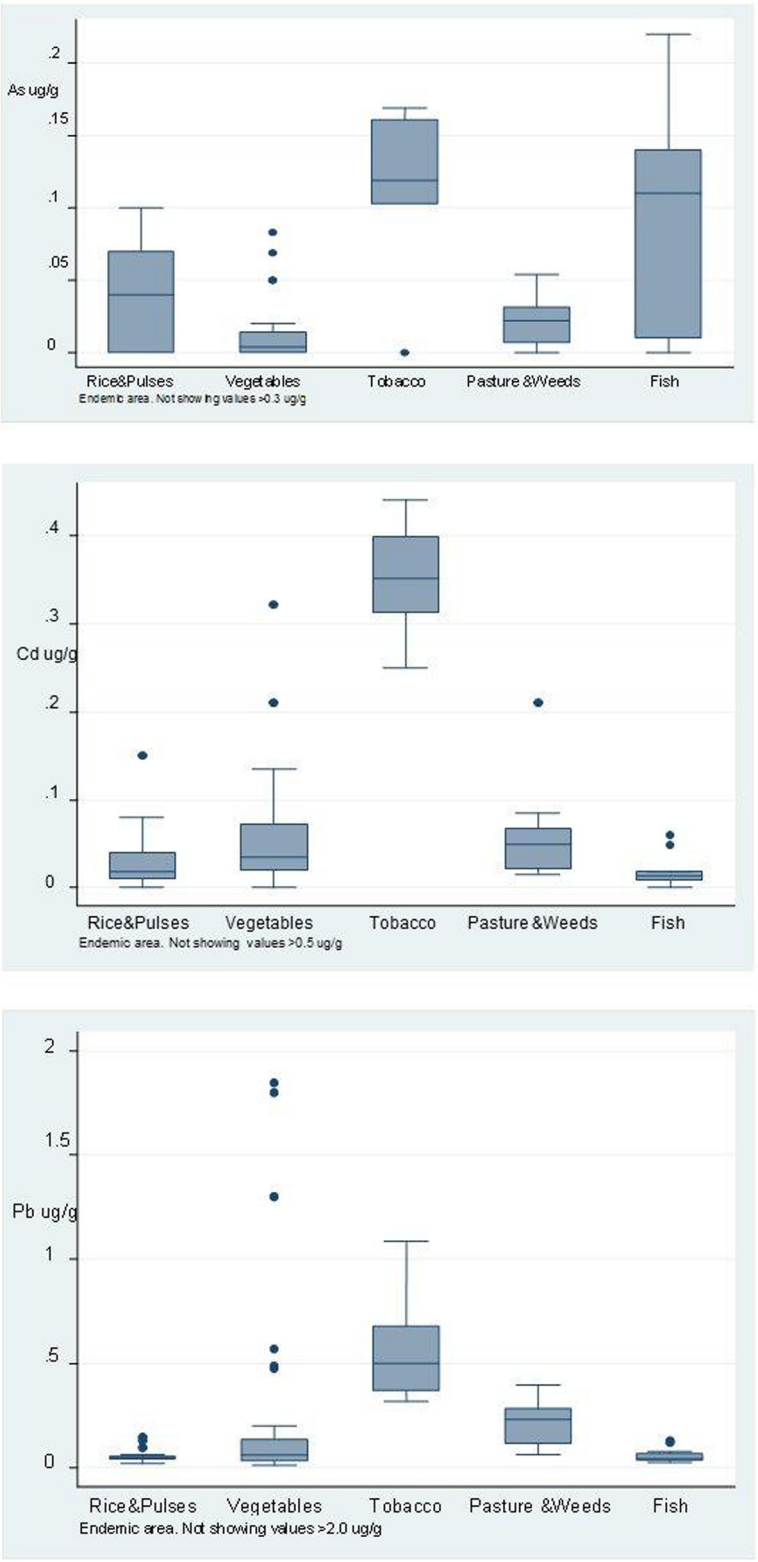
**Content of arsenic, cadmium and lead in food, tobacco leaves, pasture and weeds from the endemic area.** Horizontal lines within the boxes represent the median values. The ends of the solid lines extending on either side of the boxes represent the minimum and the maximum. The dark dots are outliers ; defined as being more than 1.5 interquartile ranges away from the box. The interquartile range is the distance between the upper part of the box and the lower part of the box. (The cadmium and lead content in certain food items exceeded the maximum stipulated reference value^a^). ^a^ The maximum levels of cadmium permitted by the Codex Alimentarius for vegetables is 0.2 mg/kg [22,23] and by the Commission of the European Communities is 0.05 mg/kg [24]. The maximum concentration of cadmium stipulated for certain types of fish by the Commission of the European Communities is 0.05 mg/kg [24]. The maximum concentration of lead stipulated for vegetables by the Commission of the European communities is 0.10 mg/kg [24].

Levels of cadmium in rice in both endemic and non-endemic areas were below the allowable limit (0.2 mg/kg; Figure 5). The maximum concentration of cadmium in vegetables in the endemic area and in the non-endemic areas was 0.322 mg/kg and 0.063 mg/kg respectively. Levels of cadmium in certain vegetables such as lotus root, and in tobacco, were high. Levels of cadmium in lotus and tobacco were higher in endemic than in non-endemic areas (lotus: mean = 0.413 mg/kg versus 0.023 mg/kg, median = 0.066 mg/kg versus 0.023 mg/kg, maximum = 1.50 mg/kg versus 0.03 mg/kg; tobacco: mean = 0.351 mg/kg versus 0.316 mg/kg, median = 0.351 mg/kg versus 0.316 mg/kg, maximum = 0.44 mg/kg versus 0.351 mg/kg in endemic versus non-endemic areas respectively).

### Arsenic, cadmium and lead in soil and agrochemicals in the endemic and non-endemic areas

The level of cadmium in surface soil in the endemic area (*n* = 94, excluding samples from reservoirs), was 1.16 μg/g compared to 0.49 μg/g in the non-endemic area (*n* = 45, excluding samples from reservoirs) (Additional file 2).

### Pesticide residues in urine

Pesticide residues were detected in the urine from individuals with CKDu (*n* = 57). The frequency of detection of 2,4-D, 3,5,6-trichloropyridinol, *p*-nitrophenol, 1-naphthol, 2-naphthol, glyphosate, AMPA was 33%, 70%, 58%, 100%, 100%, 65% and 28% respectively). Isopropoxyphenol, 2,4,5-trichlorphenol and pentachlorphenol were below detection limits. The proportions of CKDu cases with pesticide levels above reference values are shown in Table 4.

**Table 4:** **Pesticide residues in urine of CKDu cases (*n*** **= 57)**

**Parent compound**	**Biomarker**	**Reference limit (μg/l)**	**CKDu cases (μg/l), (minimum, maximum)**	**CKDu cases above reference limit (%)**
2,4-D	2,4-D	<0.3	0.5, 0.62	3.5
Pentachlorophenol	Pentachlorophenol	<2	0.3, 2.2	1.7
Chlorpyrifos	3,5,6-trichloropyridinol	<11.3	0.5, 34.7	10.5
Parathion	*p*-nitrophenol	<25	0.5, 8.88	0
Carbaryl naphthalene	1-naphthol	<19.7	0.5, 45.1	10.5
Naphthalene	2-naphthol,	<17.1	0.94, 47.88	10.5
Glyphosate	Glyphosate	<2	0.075, 3.36	3.5
Glyphosate	AMPA	<0.5	0.075, 2.65	14

## Discussion

The prevalence of CKDu found in this study (females 16.9%, males 12.9%) was higher than that reported previously (2% to 3%) [20]. Although the prevalence in females was higher, more severe stages of CKDu were seen more often in males. The reason for this discrepancy is not clear. Factors such as low iron stores in females in lower socioeconomic groups may have an influence on the excretion of heavy metals and oxidative stress on the kidney. This would make the kidneys more vulnerable to CKDu, resulting in a higher prevalence in females. On the other hand, male sex has been reported to be a risk factor for progression to end-stage renal disease [27], and this may partly explain the occurrence of more severe stages of CKDu in men.

Previous studies have reported a family history of chronic kidney disease, ayurvedic treatment, and history of snake bite as significant predictors for CKDu [10,12,13]. In the present study, older age, being female and being a chena cultivation farmer increased the risk of CKDu. Family history was positive in one-fifth of those with CKDu, and a history of snake bite was one of the exclusion criteria. Long-term use of herbal medicines or analgesics was reported in only a very small percentage of those with CKDu. Fanconi syndrome and other hereditary kidney diseases have not been reported in communities in this region.

Previous studies have reported divergent information on the role of cadmium in the causation of CKDu [14,15,19,20]. In the present study, individuals with CKDu excreted significantly higher levels of cadmium compared to those in the control group, in both the endemic and non-endemic areas. Controls in the endemic area compared to those in the non-endemic area also had significantly higher urinary excretion of cadmium. The sensitivity and specificity for urine cadmium were 80% and 53.6% respectively (AUC = 0.682, cut-off value ≥0.23 μg/g). There was a dose–effect relationship between the concentration of cadmium in urine and the stage of CKDu. A significantly higher cadmium concentration was also seen in the nails of those with CKDu compared to controls from the endemic area. Cadmium is a known nephrotoxin and urinary excretion of cadmium is considered to be a reliable indicator of cumulative long-term exposure to cadmium [6]. The mean urine concentration of cadmium in CKDu cases was above the levels demonstrated in recent studies to cause oxidative stress and decreased glomerular filtration rate and creatinine clearance [28-33]. The results of this study indicate that cadmium exposure is a risk factor for CKDu.

The mean urine concentration of arsenic in CKDu cases was also above levels known to cause oxidative injury to the kidney [33]. In CKDu cases and controls from the endemic area, concentrations of arsenic in urine and in fingernails were higher than those reported in people living in low-exposure environments [34,35]. Urine is a major pathway for excretion of arsenic from the human body, so urine levels reflect exposure. In some studies, markers of oxidative stress have been demonstrated at urine arsenic concentrations as low as 3.95 μg/g [36]. The level of total arsenic in urine is associated with chronic kidney disease in a dose–response relationship, especially when the level is greater than 20.74 μg/g [36]. These findings support the contention that chronic exposure to low levels of cadmium may be a causative factor for CKDu in Sri Lanka. Co-exposure to cadmium and arsenic is known to produce additive effects on the kidney that are more pronounced than exposure to either metal alone [37,38].

Selenium has been shown to protect the kidney from oxidative stress [39]. A selenium concentration of 80–95 μg/l is needed to maximise the activity of the antioxidant enzyme glutathione peroxidase and selenoproteins in plasma [40,41]. In this context, it is significant that serum selenium was below 80 μg/l in 38% and below 90 μg/l in 63% of individuals with CKDu. Low selenium levels may have been a contributory factor increasing the vulnerability of the kidneys to oxidative damage caused by heavy metals and metalloids.

The association of raised serum strontium levels with raised serum cadmium levels has been reported previously [42]. Strontium levels were not analysed in food or water. The most likely explanation is an alteration of strontium handling and excretion, owing to the effect of cadmium on renal tubular function.

Cadmium levels have previously been reported to be high in water sources in the domestic environment of people with CKDu, and 10–20 times the maximum stipulated level have been found in reservoirs in the endemic area [15]. The results of this study did not show this to be the case. On the contrary, the cadmium content in all water samples analysed was within normal limits, except in one sample from a reservoir that had a borderline cadmium level (3.45 μg/l).

Drinking water is a major pathway for entry of inorganic arsenic into the human body. The arsenic content in 99% of water samples was below the WHO reference value of 10 μg/l [21]. However, it has recently been suggested that the concentration of arsenic in drinking water should be no more than 5 μg/l [43].

CKDu occurs in areas where groundwater is the main source of drinking water. Groundwater in this region is known to have a high content of fluoride and calcium. People living in the region for generations have used groundwater for drinking without ill effects. However, hardness of water, the high fluoride content, poor access to drinking water and inadequate intake of water in a warm climate may influence the body burden and/or the excretion of heavy metals and oxidative damage to the kidneys caused by heavy metals.

The maximum level of cadmium for vegetables permitted by the Codex Alimentarius is 0.2 mg/kg [22,23] and the level permitted by the Commission of the European Communities is 0.05 mg/kg [24]. The maximum levels in certain vegetables grown in the endemic area exceeded these safety levels. The maximum concentration of cadmium in fish (0.06 μg/g) also exceeded the European maximum limit of 0.05 mg/kg stipulated for certain types of fish [24]. The maximum level of lead in vegetables permitted by the Commission of the European Communities is 0.10 mg/kg [24]. The maximum level of lead in vegetables in the endemic area (0.476 mg/kg) exceeded this cut-off value. Levels of cadmium and lead in vegetables and cadmium in freshwater fish from the endemic area are above the maximum levels stipulated by certain Food Safety Authorities [22-24,44].

A provisional tolerable weekly intake (PTWI) for cadmium of 7 μg/kg body weight was established by the Joint Food and Agriculture Organization of the United Nations (FAO)/WHO Expert Committee on Food Additives (JECFA) [45]. In 2011, the JECFA revised the PTWI for cadmium to 5.8 μg/kg body weight [46]. More recently, the PTWI for cadmium has been lowered to 2.52 μg cadmium/kg body weight, in order to ensure a high level of protection of all consumers, including exposed and vulnerable subgroups of the population [44]. Since the cadmium content of certain food items in the endemic area is above stipulated levels, the total weekly intake of cadmium in people living in the endemic area could exceed these safe limits, with detrimental effects on the kidneys, particularly in vulnerable people and those with predisposing factors.

Reported mean dietary exposure to inorganic arsenic in the United States of America (USA) and various European and Asian countries ranges from 0.1 to 3.0 μg/kg body weight per day [45]. Recently, the PTWI for arsenic (0.015 mg/kg body weight per week) was withdrawn and environmental authorities are in the process of collecting more data for exposure assessment [46]. The current recommendation is that every effort should be made to keep concentrations of arsenic as low as reasonably possible. The PTWI for lead is set at 0.025 mg/kg body weight per week [45].

Previous studies have reported high levels of cadmium in fertilizer (mean 47 μg/g) [15]. The maximum cadmium, lead and arsenic concentrations in phosphate fertilizer from the endemic area in the present study were 30.8 μg/g, 823.4 μg/g and 0.19 μg/g respectively. The maximum acceptable levels for cadmium, lead and arsenic, in phosphate fertilizer product, at 1% of the nutrient level, are 4 μg/g, 20 μg/g and 2 μg/g, respectively [47].

The mean concentration of cadmium in soil from the endemic area was 0.4 μg/g. Surveys of agricultural soils in the USA and Sweden have reported lower soil cadmium levels (0.265 mg/kg and 0.23 mg/kg respectively) [48,49]. The concentration of cadmium, arsenic and lead in soil, and their impact on body burden and excretion, is known to be influenced by many environmental factors such as the pH of soil, buffering capacity, content of organic matter and water quality, among others [50-52]. Cadmium accumulation by plants, for example, is influenced by the reactive soil cadmium content and pH. It is decreased by high cation exchange capacity of the soil and increased by higher soil temperature [49-52]. The hardness and high content of fluoride in water in the endemic area may also influence the dynamics of cadmium in soil, absorption by plants [17] and excretion from the kidney.

Certain pesticide residues were above reference levels in 31.6% of CKDu cases. Residues are demonstrative of the extent of the environmental distribution of pesticides and certain pesticides are known to be nephrotoxic [4,5,53]. Simultaneous exposure of people to heavy metals and nephrotoxic pesticides may be a contributory factor in the pathogenesis and progression of CKDu.

Compared to previous studies conducted on CKDu, the present study has several strengths: (i) use of a consistent case definition for CKDu; (ii) analysis of a range of biological samples from individuals with CKDu; (iii) comparison of control groups within and outside the endemic area; and (iv) use of sensitive analytical techniques. Studies conducted hitherto to determine the prevalence and aetiology of CKDu [10,12,13,16,18,20] have relied on dipstick urinalysis to identify kidney disease. The present study is also the first in which heavy metals, metalloids and other elements in environmental and biological samples and pesticide residues in urine have been analysed.

There are several limitations in the study. Other kidney disease such as HIV nephropathy could fulfil the case-definition criteria used for CKDu. As HIV is not prevalent in Sri Lanka, it was not excluded through blood tests. The presence of glomerulonephritis was not excluded by biopsy but was based on past medical records and diagnosis cards. The sensitivity and specificity of the case definition relative to biopsy-proven CKDu is also not known. Stage 1 CKDu is defined by persistent microalbuminuria and may overestimate the prevalence of CKDu. The case definition required albuminuria. As a result, people with CKDu who have a low eGFR and no albuminuria were excluded from the study. In addition, the CKD-EPI equation used to estimate eGFR [25] has not been validated in people from South Asia. It is not known whether the albuminuria of CKDu responds to treatment for high blood pressure. If it does, an individual could then be excluded based on their ACR, despite having the disease.

CKDu has been reported in other populations as well [54-57]. Lessons learnt from other countries demonstrate that sound public health policies to ensure access to safe drinking water; regulatory control to ensure appropriate use of agrochemicals including fertilizer; hazardous waste remediation; regulatory control to prevent pollution of the environment from discarded batteries containing heavy metals; tobacco control; and reduction of air pollution can reduce exposure to heavy metals [58,59]. Based on the findings of this study, the Government and the Ministry of Health of Sri Lanka have already initiated multisectoral collaborative action with the Ministries of Agriculture, Irrigation, Scientific Affairs and Social Services, to mitigate the exposure of people to environmental nephrotoxic substances. Steps are being taken to strengthen the water supply scheme in the endemic area as well as the regulations related to procurement and distribution of fertilizers and pesticides. Further studies are ongoing to investigate the contributory role of infections in the pathogenesis of CKDu.

## Conclusions

The results of this cross-sectional study indicate that multiple agents may play a role in the pathogenesis of CKDu. Herbal medicines and indiscriminate use of analgesics are unlikely to be causative factors of CKDu. Results show chronic exposure of people in the endemic area to low levels of cadmium through the food chain and also to pesticides. They may also be exposed to lead and arsenic through the food chain. Urine concentrations of cadmium and arsenic in individuals with CKDu were at levels known to cause kidney damage. Significantly higher urinary excretion of cadmium in individuals with CKDu, and the dose–effect relationship between urine cadmium levels and CKDu stages, indicate that cadmium is a risk factor for the pathogensis of CKDu in Sri Lanka. Deficiency of selenium and genetic susceptibility seen in individuals with CKDu suggest that they may be predisposing factors for the development of CKDu when people are exposed to nephrotoxins.

## Abbreviations

ACR: Albumin–creatinine ratio; AMPA: Aminomethylphosphonic acid; AUC: Area under the receiver operating characteristic curve; CI: Confidence interval; CKD-EPI: Chronic kidney disease epidemiology collaboration; CKDu: Chronic kidney disease of uncertain aetiology; CV: Coefficient of variation; eGFR: Estimated glomerular filtration rate; FAO: Food and agriculture organization of the United Nations; GC-MS: Gas chromatography-mass spectroscopy; HbA1c: Glycosylated haemoglobin; ICP-MS: Inductively coupled plasma mass spectrometry; JECFA: Joint FAO/WHO expert committee on food additives; LC-MS: Lliquid chromatography with tandem mass spectrometry; MS: Mass spectrometry; OR: Odds ratio; PRWI: Provisional tolerable weekly intake; ROC: Receiver-operating characteristic; SD: Standard deviation; USA: United States of America; WHO: World Health Organization.

## Competing interests

None of the National Research Project Team members have declared any relationship with companies that may have a financial interest in the information contained in the manuscript.

## Authors’ contributions

SM led the development of the research proposal. She co-chaired the international advisory board and directed the execution and the technical aspects of the research project including analysis of results and drafting and finalization of the paper on behalf of the World Health Organization. FM was responsible for the implementation of the project at the country level and contributed to the paper. NJ and PM co-chaired the national CKDu research project committee on behalf of the Ministry of Health and contributed to the paper. All authors read and approved the final manuscript.

## Pre-publication history

The pre-publication history for this paper can be accessed here:

http://www.biomedcentral.com/1471-2369/14/180/prepub

## Supplementary Material

Additional file 1 Details of sample preparation and analytical techniques. Click here for file

Additional file 2: Table S1 Urine concentration of metals (sodium, potassium, calcium, magnesium, copper, zinc and titanium) in CKDu cases. **Table S2.** Serum concentration of aluminium, chromium, selenium and strontium in CKDu cases. **Table S3.** Concentration of arsenic, cadmium and lead in surface soil and in phosphate fertilizer, pesticides and weedicides, in the endemic area compared with a non-endemic area. Samples of soil from vegetable plots from the endemic area were obtained from the vicinity of households with CKDu patients. Click here for file
