# Supplementary material for: Chronic kidney disease of uncertain aetiology: prevalence and causative factors in a developing country
Source: BMC Nephrol. 2013 Aug 27;14:180. doi: 10.1186/1471-2369-14-180 (PMC3765913; doi:10.1186/1471-2369-14-180)
Supplement: Additional file 1 — Details of sample preparation and analytical techniques. [file 1471-2369-14-180-S1.doc]

# Additional file

## Specimen handling and analysis

Samples were collected in uncontaminated collection vials and stored frozen (–20°C) until transfer to the laboratory.

**Urine** and **water** samples were defrosted and an aliquot of 0.40 ml was removed and mixed with 0.05 ml Suprapur® nitric acid (HNO_3_, 65% v/v; Merck, Germany) + 0.02 ml Internal Standard (Perkin Elmer, USA) + 1.55 ml HNO_3_ (1% v/v) before analysis.

Before measurement, approximately 0.50 g of the samples of **vegetables** and **agrochemicals** were first digested in a mixture consisting of 3.0 ml of Suprapur® HNO_3_ and 9.0 ml Emsure® hydrochloric acid (HCl, 37% v/v; Merck, Germany). For the measurement of arsenic (As), an adapted digestion liquid consisting of only 8.0 ml Suprapur® HNO_3_was applied, to avoid interference of chlorine with the As measurement.

For the **soil** samples, 0.50 g was digested in a mixture consisting of 2.0 ml Suprapur® HNO_3_ + 6.0 ml Emsure® HCl + 2.0 ml Emsure® hydrofluoric acid (HF; Merck, Germany). A Milestone microwave digestion unit (Type Ethos, Analys, Belgium), was used for the digestion. Samples were digested for 30 minutes, during which temperature and pressure were increased, followed by a 3-h cooling step. Following digestion, volumes were adjusted to 20 ml or 10 ml (for adapted procedure As measurement) with Milli-Q water (Millipore Corporation, USA).

Measurements were then performed after mixing 0.40 ml of the digestion liquid with 0.02 ml internal standard and 1.60 ml HNO_3_ (1% v/v).

Samples of **hair** and **nails** of approximately 0.15 g were first washed in 0.01 v/v% Triton X-100 (Merck, Germany) and sonicated for 10 minutes. Triton X-100 was then removed and samples rinsed three times with Milli-Q water. Samples were then soaked in 10 ml Emsure® acetone (Merck, Germany) and again sonicated for 10 minutes. Acetone was removed and samples were dried in an oven.

Following the washing procedure, ±0.12 g of the dried samples were digested in 1.00 ml Suprapur® HNO_3_ for ±24 hours at 60°C in loosely stoppered polypropylene tubes until complete digestion and adjusted to 10 ml with Milli-Q water. Samples were then measured after mixing 0.40 ml of the digestion liquid with 0.02 ml Internal Standard and 1.60 ml HNO_3_ (1% v/v).

Measurements of arsenic, cadmium, lead and other elements in urine, water, vegetables, agrochemicals, soil, bone and soft tissue were performed by inductively coupled plasma mass spectrometry (ICP-MS, Varian 810, USA) equipped with an SPS 3 automatic sample preparation system (Varian).

**Serum** was defrosted and 0.20 ml was removed and diluted in 0.40 ml of a Triton X-100 (0.4% v/v)/Suprapur® HNO_3_ (0.4% v/v) mixture for measurement of aluminium, strontium and chromium.

For measurement of selenium, 0.10 ml of serum was diluted in 0.40 ml Triton X-100 (0.4%)/Suprapur® HNO_3_ (0.4%) and a Pd/Mg matrix modifier (Perkin Elmer) was used to eliminate chemical interference.

Serum analyses were performed by electrothermal atomic absorption spectrometery with Zeeman background correction (Model AAnalyst 800, Perkin Elmer, USA) and equipped with an AS 800 autosampling system (Perkin Elmer).

For quality control of ICP-MS measurements, internal standards (i.e. non-significant metals) were used to control for signal fluctuations during the analytical run. Analytical accuracy was further checked by co-digestion and co-analysis of certified reference material BCR® (Institute for Reference Materials and Measurements, Geel, Belgium). Urine and serum control samples were obtained from RECIPE (Germany). Multiple measurements of blank sample (start and end) during the analytical run were performed to control contamination and signal baseline drift.

The linearity of calibration curves was checked for each analytical run and samples with concentrations above the linear range of the curves were further diluted.

Limits of detection for arsenic, cadmium and lead in water and urine were 0.20 µg/l, 0.17 µg/l, and 0.09 µg/l respectively. Limits of detection for arsenic, cadmium and lead in digestion fluids (considering a sample weight of 0.1 g) were 40.4 ng/g, 34.8 ng/g and 18.4 ng/g respectively.

The limits of detection for aluminium, strontium, chromium and selenium were 0.1 µg/l, 0.5 µg/l, 0.01 µg/l and 1 µg/l respectively.

Analyses of metals and metalloids were done in a contract laboratory (Laboratory of Pathophysiology of the University of Antwerp, Belgium), which has a trace element external quality control scheme.

## Reliability of cadmium and arsenic assays

The within-run (intra)-coefficient of variation (CV) of different dilutions for cadmium is 10.8% for a concentration of 1.78 µg/l, while the between-run (inter)-CVs (including the whole digestion procedure, dilutions, etc,) are 9.8% for a cadmium concentration of 9.35 µg/l and 18.08% for a concentration of 0.19 µg/l.

The within-run (intra)-CV of different dilutions for arsenic is 7.2% for a concentration of 2.79 µg/l, while the between-run (inter)-CVs (including the whole digestion procedure, dilutions, etc) are 10.6% for an arsenic concentration of 4.82 µg/l and 12.6% for a concentration of 5.32 µg/l.

In view of the rather complex sample preparation required for these types of samples in which relatively low concentrations had to be measured, these values are within the acceptable range of analytical performance.

The accuracy of the arsenic measurement as assessed against BCR® reference material was 0.03 µg/g (reference: 0.03 µg/g) for hair samples, and 38.6 µg/l (reference range: 34.8–52.2 µg/l) for urine.

The accuracy of the cadmium measurement as assessed against BCR® reference material was 0.45 µg/g (reference: 0.54 µg/g) for hair samples, 0.47 µg/g (reference 0.54 µg/g) for environmental samples and 1.87 µg/l (reference range: 1.71–2.57 µg/l) for urine.

## Determination of pesticide residues in urine of people with CKDu

Urine samples were shipped on dry ice and stored at –18°C until analysis. Analysis was done by means of validated LC-MS/MS, GC-MS and GC-MS/MS methods. Quality control and calibration verifications showed good precision and accuracy throughout the analyses.
