# Supplementary material for: Chronic kidney disease of uncertain aetiology: prevalence and causative factors in a developing country
Source: BMC Nephrol. 2013 Aug 27;14:180. doi: 10.1186/1471-2369-14-180 (PMC3765913; doi:10.1186/1471-2369-14-180)
Supplement: Additional file 2: Table S1 — Urine concentration of metals (sodium, potassium, calcium, magnesium, copper, zinc and titanium) in CKDu cases. Table S2. Serum concentration of aluminium, chromium, selenium and strontium in CKDu cases. Table S3. Concentration of arsenic, cadmium and lead in surface soil and in phosphate fertilizer, pesticides and weedicides, in the endemic area compared with a non-endemic area. Samples of soil from vegetable plots from the endemic area were obtained from the vicinity of households with CKDu patients. [file 1471-2369-14-180-S2.doc]

**Additional file 2: Table S1**. Urine concentration of metals (sodium, potassium, calcium, magnesium, copper, zinc and titanium) in CKDu cases

|  | Metals in urine *n* = 107, (mg/g creatinine) | | | |
| --- | --- | --- | --- | --- |
|  | Mean | Median | Minimum | Maximum |
| Sodium | 4105.50 | 3544.00 | 425.00 | 17458.00 |
| Potassium | 917.94 | 800.00 | 243.00 | 2469.00 |
| Calcium | 80.45 | 67.00 | 4.00 | 368.00 |
| Magnesium | 79.89 | 80.00 | 2.00 | 169.00 |
| Copper | 13.34 | 11.00 | 3.70 | 91.10 |
| Zinc | 229.99 | 235.99 | 31.00 | 510.00 |
| Titanium | 0.26 | 0.24 | 0.03 | 0.88 |

**Additional file 2: Table S2.** Serum concentration of aluminium, chromium, selenium and strontium in CKDu cases

|  | Serum concentration (µg/l), *n* = 171 | | | |
| --- | --- | --- | --- | --- |
|  | Mean | Median | Minimum | Maximum |
| Aluminium | 4.13 | 3.00 | 1.00 | 12.00 |
| Chromium | 0.118 | 0.06 | 0.01 | 1.15 |
| Selenium^a^ | 88.27 | 84.5 | 50.0 | 121.8 |
| Strontium^b^ | 83.17 | 82.00 | 29.00 | 198.00 |

^a^ Serum selenium reference range 54–163 µg/l.

^b^ Serum strontium reference range 14–84 µg/l.

**Additional file 2: Table S3.** Concentration of arsenic, cadmium and lead in surface soil and in phosphate fertilizer, pesticides and weedicides, in the endemic area compared with a non-endemic area. Samples of soil from vegetable plots from the endemic area were obtained from the vicinity of households with CKDu patients.

| Area and source | Arsenic µg/g | Cadmium µg/g | Lead µg/g |
| --- | --- | --- | --- |
|  | Mean, median (minimum, maximum) | | |
| Endemic area |  |  |  |
| Soil from paddy cultivation  *n*= 45 | 0.16, 0.11, (0.00, 0.85) | 0.49, 0.43 (0.16, 0.56) | 16.54, 15.75 (5.03, 34.54) |
| Soil from chena (shifting) cultivation *n*= 20 | 0.06, 0.04 (0.00, 0.22) | 0.40, 0.36 (0.17, 1.27) | 15.41, 13.82 (8.25, 28.33) |
| Soil from vegetable plot (home) *n*= 23 | 0.11, 0.07 (0.00, 0.46) | 3.48, 0.37 (0.16, 70.00) | 17.46, 16.76 (6.69, 41.02) |
| Soil from crop land *n*= 6 | 0.05, 0.06 (0.00, 0.01) | 0.60, 0.5 (0.17, 1.47) | 20.55, 20.29 (9.98, 32.1) |
| Soil from reservoirs *n*= 6 | 0.60, 0.5 (0.17, 0.43) | 0.66, 0.52 (0.15, 1.36) | 19.16, 17.16 (7.11, 33.49) |
| Phosphate fertilizer  *n*= 13 | 0.06, 0.04 (0.00, 0.19) | 2.98, 0.04 (0.01, 30.79) | 94.23, 1.42 (0.17, 823.41) |
| Weedicides and/or pesticides *n*= 26 | 6.73, 1.68 (0.01, 94.93) | 0.77, 0.31 (0.05, 9.34) | 40.62, 1.79 (0.83, 930.81) |
| Non-endemic area |  |  |  |
| Soil from paddy cultivation *n*= 21 | 0.17, 0.08 (0.01, 0.99) | 0.45, 0.40 (0.01, 1.61) | 14.49, 16.95 (0.02, 39.95) |
| Soil from chena (shifting) cultivation *n*= 10 | 0.40, 0.29 (0.09, 1.57) | 0.59, 0.55 (0.34, 0.93) | 14.84. 13.93 (5.42. 26.1) |
| Soil from vegetable plot (home) *n*= 10 | 0.27, 0.24 (0.08, 0.53) | 0.47, 0.41 (0.29, 0.84) | 18.01, 18.03 (5.57, 32.87) |
| Crop land *n*= 4 | 0.13, 0.13 (0.09. 0.18) | 0.28, 0.28 (0.24, 0.33) | 7.96, 7.96 (3.15, 12.77) |
| Phosphate fertilizer *n*= 5 | 0.43, 0.19 (0.00, 1.22) | 0.49, 0.03 (0.01, 1.28) | 20.29, 0.65 (0.09, 98.52) |
| Weedicides and pesticides *n*= 8 | 3.81, 1.38 (0.01, 13.15) | 0.76, 0.3 (0.05, 2.0) | 15.65, 1.89 (1.01, 56.39) |
